# CA125 as a predictor of endometrial cancer lymphovascular space invasion and lymph node metastasis for risk stratification in the preoperative setting

**DOI:** 10.1038/s41598-022-22026-1

**Published:** 2022-11-17

**Authors:** K. Shawn LyBarger, Hunter A. Miller, Hermann B. Frieboes

**Affiliations:** 1 Sarah Cannon Cancer Institute, HCA MidAmerica, HCA Midwest Health, Kansas City, MO 64132 USA; 2 Department of Obstetrics and Gynecology, University of Missouri, Kansas City, MO USA; 3 Department of Bioengineering, University of Louisville, Louisville, KY USA; 4 Department of Pharmacology and Toxicology, University of Louisville, Louisville, KY USA; 5 UofL Health – Brown Cancer Center, University of Louisville, Louisville, KY USA; 6 Center for Predictive Medicine, University of Louisville, Louisville, KY USA

**Keywords:** Endometrial cancer, Surgical oncology

## Abstract

Endometrial cancer (EC) is the most common malignancy of the female reproductive system. Cancer antigen 125 (CA125) is a serum tumor marker widely reported in EC patients, particularly those with poor prognostic factors such as grade 3 tumors, deep myometrial invasion, lymph node metastasis (LNM), and extra-uterine disease. This retrospective study stratifies pre-operative CA125 levels to evaluate odds ratios (OR) and relative risk (RR) between CA125 levels and the likelihood of +LNM, lymphovascular space invasion (LVSI), grade, and stage. Patient charts for women 18 years or older with a diagnosis of EC and pre-operative or within one week CA125 measurement from January 2000 to January 2015 at a regional hospital were reviewed. OR and RR were determined by unconditional maximum likelihood estimation for CA125 levels as the predictor with staging, grade, +LVSI and +LNM as outcomes. The largest increase in risk for patients having stage I/II/III disease was 52% greater (1.52-fold risk) while largest increase in risk for patients having stage III/IV disease was 67% greater (1.67-fold risk), both at CA125 ≥ 222U/ml. Patients with CA125 ≥ 122U/ml had significantly increased risk of +LNM, with maximum increase in risk of 98% (1.98-fold risk) at 222U/ml. Patients with CA125 ≥ 175U/ml had significantly increased risk of +LVSI, with maximum increase in risk of 39% (1.39-fold risk) at 222U/ml. This study shows that elevated CA125 levels correspond to increased stage, +LVSI, and +LNM in patients with EC.

## Introduction

Endometrial cancer (EC) is the most common malignancy of the female reproductive system in developed countries. According to the American Cancer Society, approximately 66,570 new cases of cancer of the body of the uterus were diagnosed in 2021, and almost 12,940 women will die from the disease^1^. A rising incidence of EC has been reported partly due to the obesity epidemic in the United States^2^. Consequently, the number of women diagnosed with EC is expected to significantly increase in coming years.

The identification of those at highest risk of recurrence is vital in treatment planning. Since 1998 the International Federation of Gynecology and Obstetrics (FIGO)^3^ has undergone several iterations of the surgical staging of EC. In an effort to achieve postoperative pathological staging, the primary surgical procedure for patients with all stages is a total hysterectomy with bilateral salpingo-oophorectomy and some form of lymph node sampling, whether sentinel lymph nodes or systematic lymphadenectomy. The addition of pelvic and or para-aortic lymph node dissection depending on various factors, such as tumor size, grade, subtype, myometrial invasion (MI), lymphovascular space invasion (LVSI), cervical involvement, and extrauterine metastasis is also performed^4,5^. Despite this, extensive lymphadenectomy has been linked to an increased incidence of surgical related complication such as lymphedema, deep vein thrombosis (DVT), intraoperative hemorrhage, and lymphocyst formation^5^. Further, lymph node sampling does not necessarily improve survival^6^. Several studies have argued whether a lymphadenectomy should be performed in patients with tumor size < 2 cm, tumor grade 1 or 2, endometrioid subtype, and < 50% myometrial invasion^7^, since the chance of lymph node metastasis (LNM) is < 5%^8^. While routine lymphadenectomy does not improve disease-free or overall survival, positive lymph nodes do aid in the prognosis and management of the disease. Knowledge of patients with a higher risk of lymph node involvement preoperatively would be beneficial when planning for surgery and discussing prognosis and surgical procedure with patients.

The use of cancer antigen 125 (CA125) as a predictor of endometrial cancer lymphovascular space invasion and lymph node metastasis remains understudied. CA125 is a serum tumor marker widely used in epithelial ovarian cancers^9^. Ever since it was found that serum CA125 levels increased with the clinical course of some malignancies^10^, its use as a tumor marker has been reported in EC patients, particularly those with poor prognostic factors such recurrences, grade 3 tumors, deep myometrial invasion, LNM, and extrauterine disease. Previous work has evaluated preoperative serum CA125 levels as a predictor of metastatic disease and survival^11,12^. While studies have attempted to stratify CA125 utility, its clinical value in the evaluation of EC remains ambiguous since it can be mildly elevated in other non-malignant conditions^13,14^. Its use either in combination with imaging or alone may be able to help predict or stratify patients in a preoperative setting. Further, the marker is considered partially helpful in evaluating patients with significant comorbidities and perioperative risk who may not be candidates for comprehensive staging^15^. Additionally, CA125 has been incorporated into several preoperative predictive models with varying degrees of success. Clinically, CA125 can be a sensitive marker when using a cutoff of 35U/ml since only around 1% of healthy women have greater levels^16^. Furthermore, 6% of women with benign disease and 23% of women with no gynecological cancers have levels ≥ 35U/ml^16^, with elevated levels in over 80% of women with non-mucinous ovarian cancer.

Ideally, to minimize overtreatment and complications following a lymphadenectomy in EC patients, risk factors for + LNM could be used to distinguish patients more likely to benefit from lymphadenectomy versus those who would not. Therefore, the purpose of this study was to identify odds ratios (OR) and relative risks (RR) between pre-operative CA125 levels and the likelihood of + LNM, + LVSI, high grade, and advanced stage.

## Results

**Figure 1:**
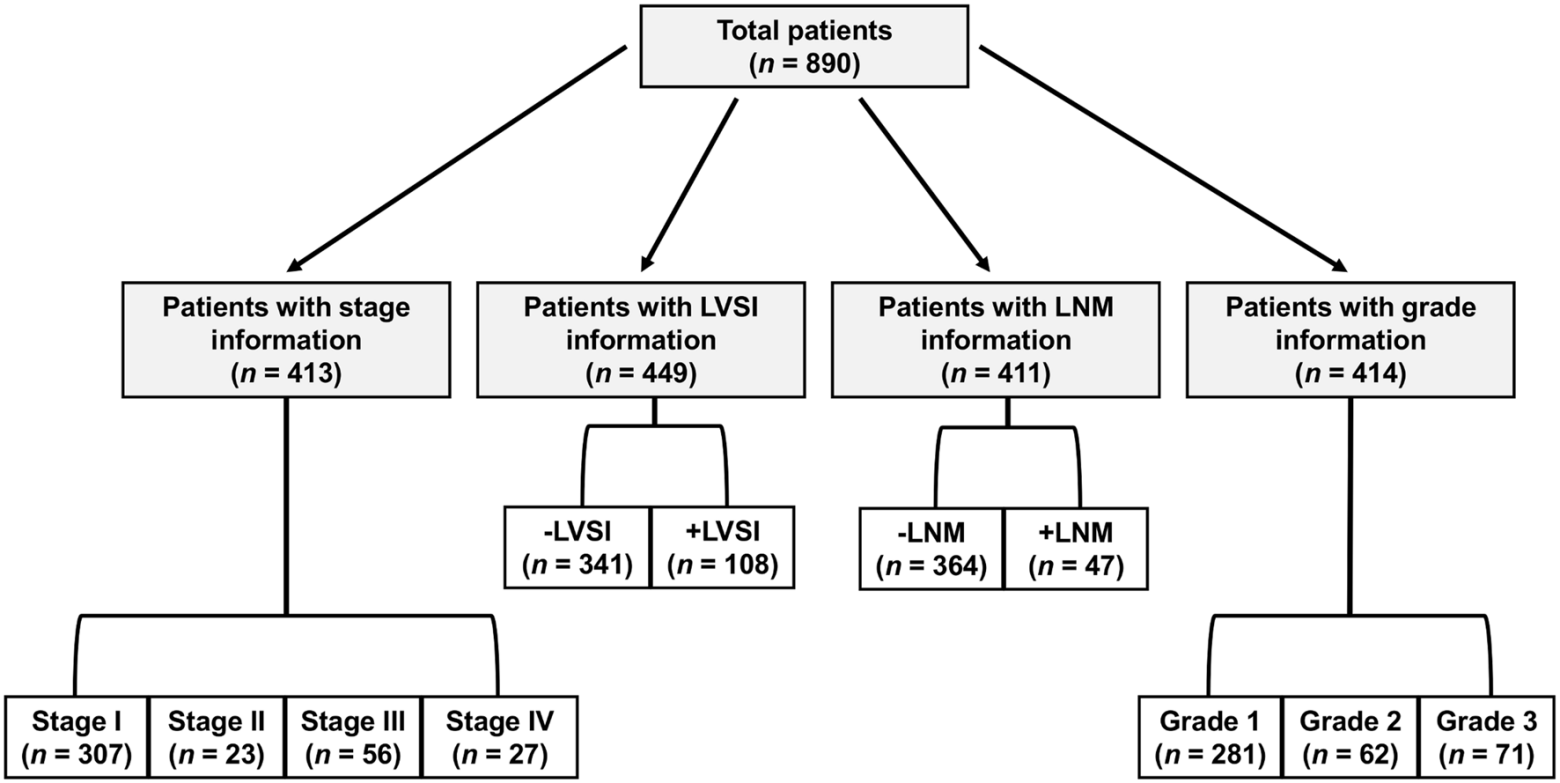
Classification of patient population based on outcome measures. Patients within each group are not mutually exclusive, with 342 patients having information available for each of the four groups. LVSI = lymphovascular space invasion; LNM = lymph node metastasis.

A total of 890 patients with a diagnosis of endometrial cancer were included in the primary analysis, out of which 413 had pre-operative staging information, 414 had pre-operative grade information, 449 had LVSI information, and 411 had LNM information (Fig. 1). Patient statistics are summarized in Table 1.

**Table 1:** Summary statistics of patient population.

	Patient population (n = 890)					
	With staging information	CA125 ≤ 35	CA125 > 35	Average age at diagnosis	Median CA125 (U/ml)	Average CA125 (U/ml)
Total	413 (46.4% of patients)	340 (82.3%)	73 (17.7%)	62.4 (61.3–63.5)	12 (11–13)	66.0 (39.22–101.79)
Stage I	307 (34.5%)	289 (94.1%)	18 (5.9%)	61.5 (60.2–62.8)	10 (9–11)	15.8 (13.1–19.3)
Stage II	23 (2.6%)	16 (69.6%)	7 (30.4%)	68.5 (63.9–73.0)	13 (7–33)	24.2 (14.8–33.6)
Stage III	56 (6.3%)	27 (48.2%)	29 (51.8%)	63.4 (60.2–66.6)	38.5 (23.0–58.5)	109.6 (67.2–159.5)
Stage IV	27 (3.0%)	8 (29.6%)	19 (70.4%)	65.1 (61.6–68.7)	98 (37–175)	582.3 (197.9–1069.2)
	**With LVSI information**					
Total	449 (50.5% of patients)	365 (81.3%)	84 (18.7%)	62.8 (61.7–63.8)	12 (11–13)	66.6 (39.5–99.83)
−LVSI	341 (76.0%)	307 (90.0%)	34 (10.0%)	62.3 (61.19–63.5)	11 (9–11)	31.3 (19.6–45.9)
+LVSI	108 (24.0%)	58 (53.7%)	50 (46.3%)	64.2 (61.9–66.4)	32.5 (24–40)	178.1 (81.1–316.8)
	**With LNM information**					
Total	411 (46.2% of patients)	341 (83.0%)	70 (17.0%)	62.7 (61.6–63.7)	12 (11–13)	53.3 (34.9–76.9)
−LNM	364 (88.6%)	324 (89.0%)	40 (11.0%)	62.6 (61.5–63.8)	11 (10–12)	36.6 (21.4–58.9)
+LNM	47 (11.4%)	17 (36.2%)	30 (63.8%)	63.1 (59.8–66.4)	55 (34–83)	184.4 (97.8–297.4)
	**With grade information**					
Total	414 (46.5% of patients)	341 (82.5%)	73 (17.5%)	62.7 (61.7–63.8)	12 (11–13)	62.5 (36.43–99.7)
Grade 1	281 (67.8%)	253 (90.0%)	28 (10.0%)	62.1 (60.8–63.3)	11 (10–12)	23.5 (17.3–31.3)
Grade 2	62 (15.0%)	52 (83.9%)	10 (16.1%)	63.8 (60.7–66.8)	12 (9.0–13.5)	30.2 (17.8–45.7)
Grade 3	71 (17.1%)	36 (50.7%)	35 (49.3%)	64.7 (62.1–67.4)	34 (20–52)	244.9 (104.9–432.6)

*LVSI* Lymphovascular space invasion, *LNM* Lymph node metastasis. 95% CI is shown in parentheses for Average Age at Diagnosis, Median CA125, and Average CA125.

**Figure 2:**
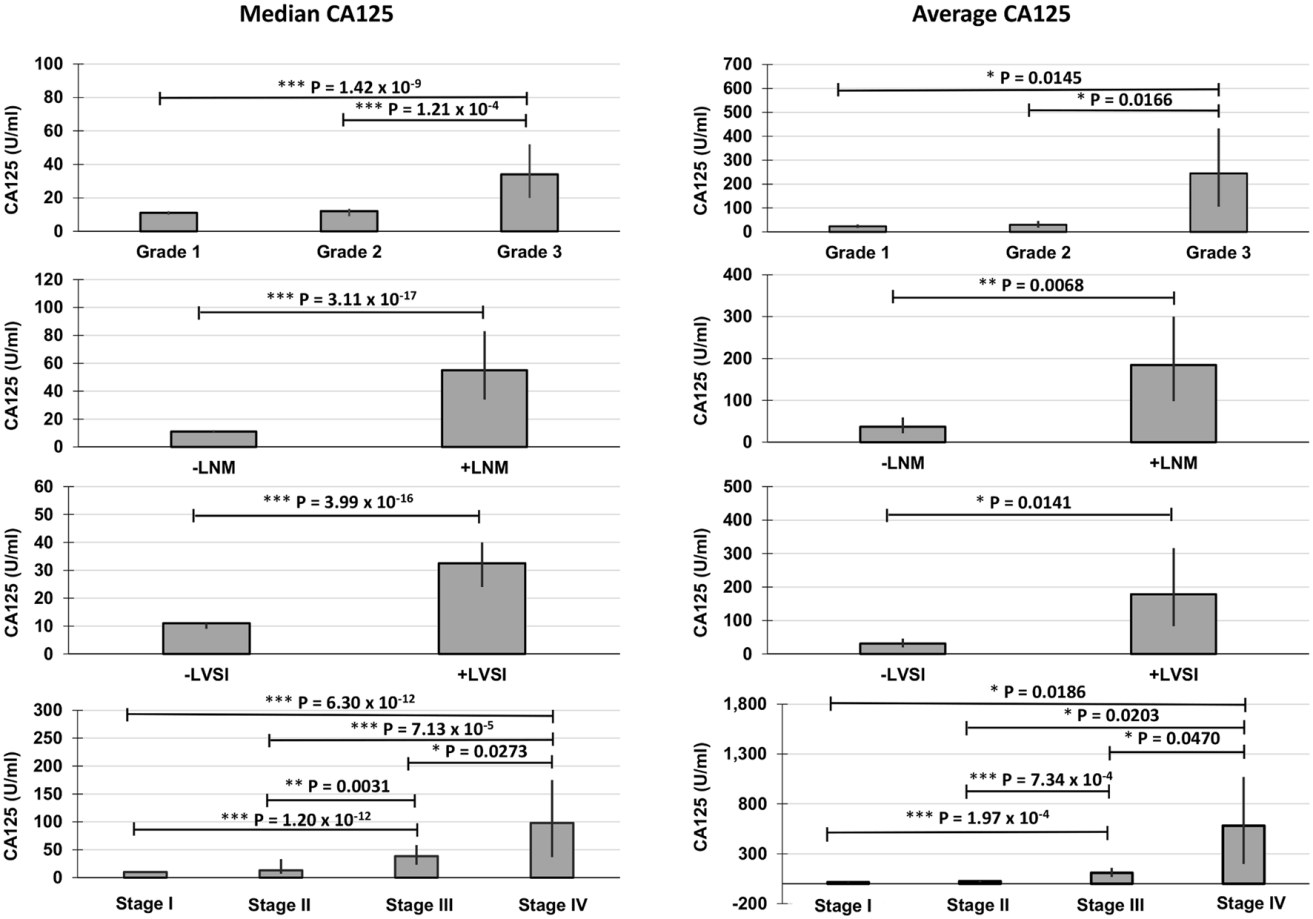
Median CA125 (U/ml) (left) and average CA125 (U/ml) (right) for all patient classifications. LVSI = lymphovascular space invasion; LNM = lymph node metastasis. Bars represent 95% CI. For medians, a Wilcoxon rank-sum test was used for statistical significance. For averages, a student’s T-test was used. *p ≤ 0.05; **p ≤ 0.01; ***p ≤ 0.001.

Median and average CA125 levels across all groups is shown in Fig. 2. There is a significant increase in both median and average CA125 for + LNM and + LVSI. Additionally, CA125 generally increased as stage and grade increased.

**Figure 3:**
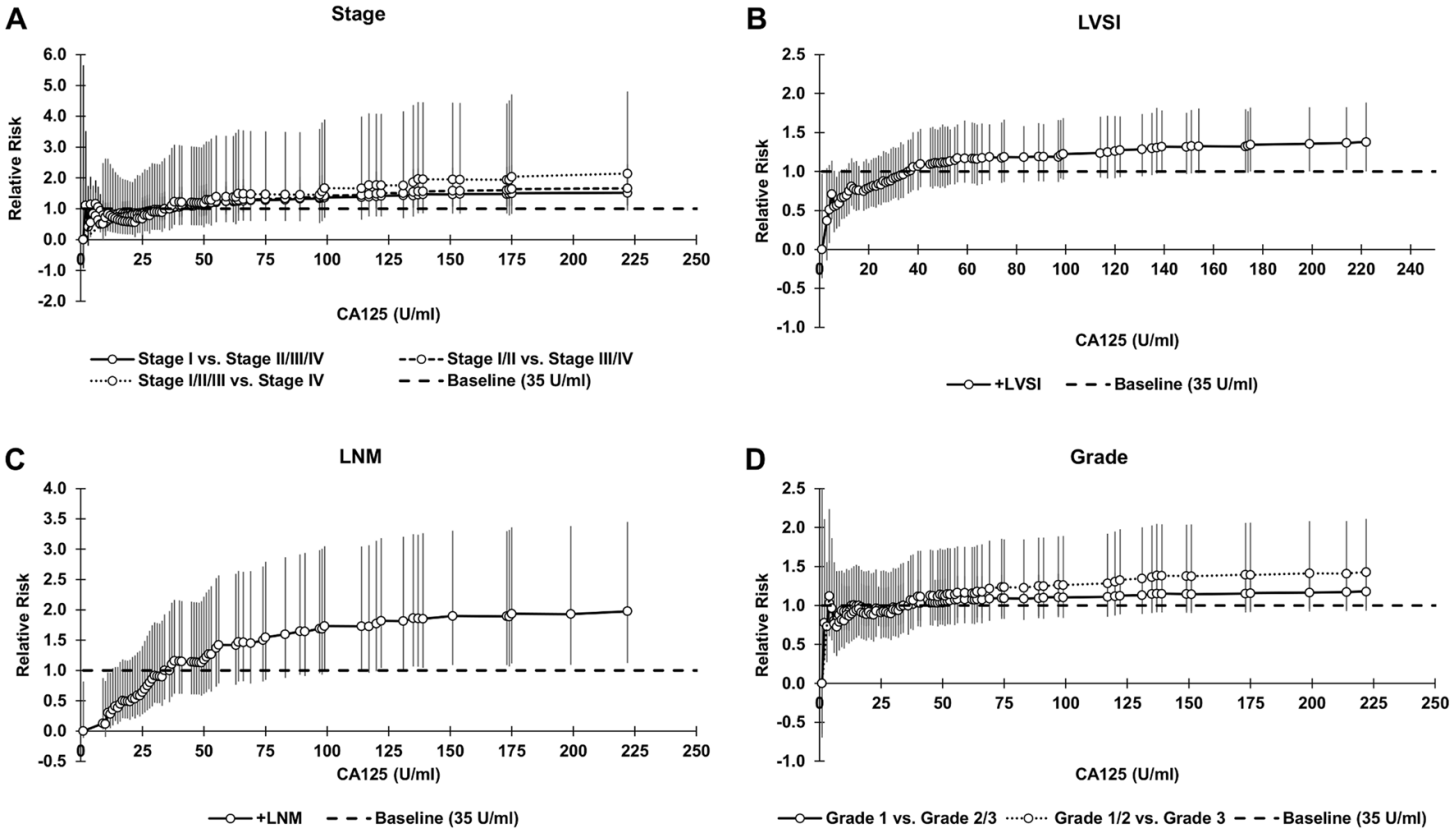
Relative risk (RR) for all CA125 thresholds from 1 to 222. RR for baseline CA125 of 35U/ml is equal to 1.0. *P* values for OR and RR were calculated with Fisher's exact test using a two-sided 95% CI, where the CI was calculated using a normal approximation. Bars represent 95% CI.

**Figure 4:**
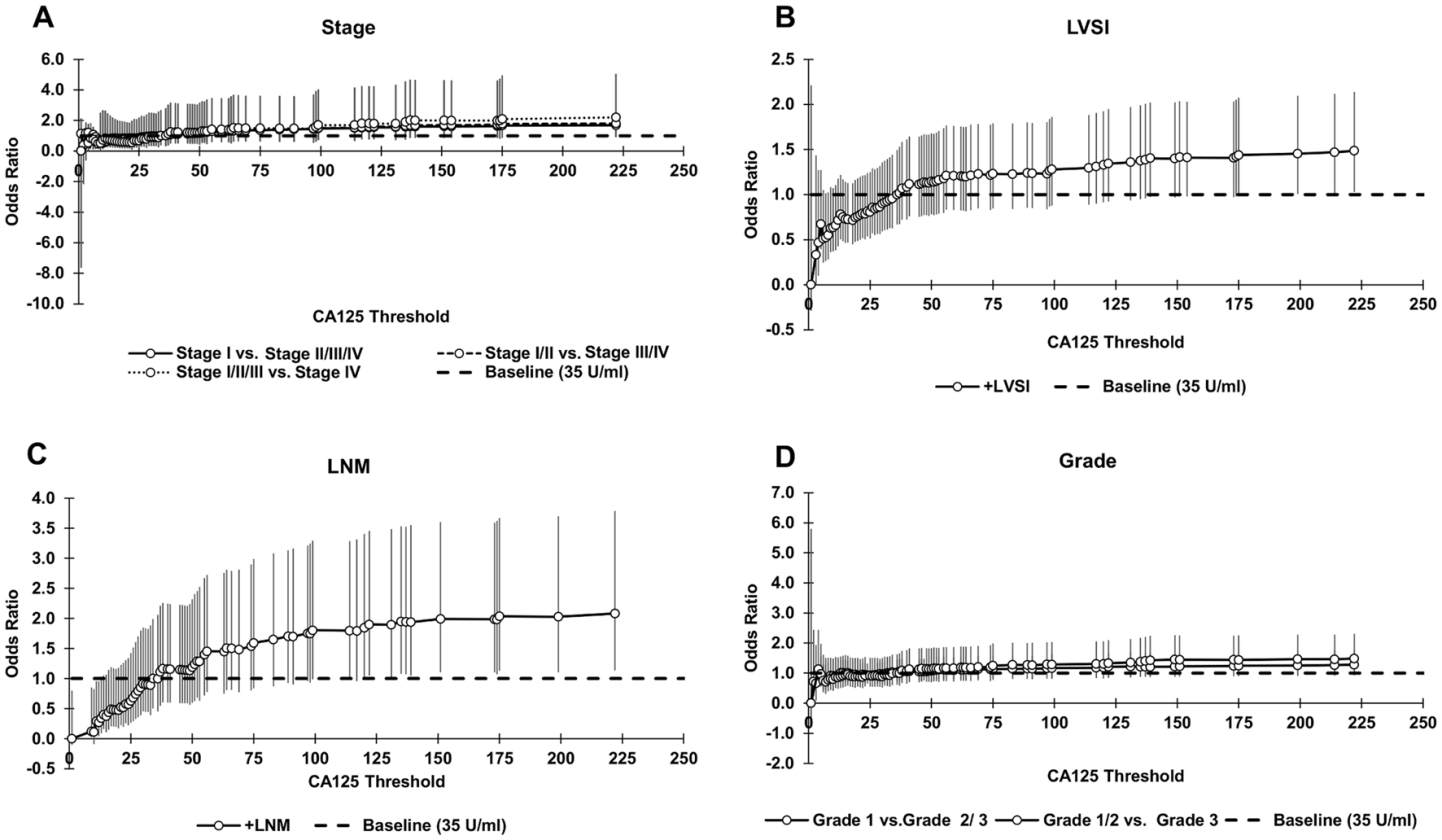
Odds ratio (OR) for all CA125 thresholds from 1 to 222. OR for baseline CA125 of 35U/ml is equal to 1.0. *P* values for OR and RR were calculated with Fisher's exact test using a two-sided 95% CI, where the CI was calculated using a normal approximation. Bars represent 95% CI.

RR and OR trends between CA125 levels and all patient classifications are shown in Figs. 3 and 4, respectively. Generally, RR and OR at a CA125 threshold below 35U/ml was associated with a better outcome (0 $\le$ RR $<$ 1, 0 $\le$ OR $<$ 1) and above 35U/ml was associated with a worse outcome (RR $>$ 1, OR $>$ 1). RR and OR tended to increase above a CA125 threshold of 35U/ml for all patient classifications.

**Figure 5:**
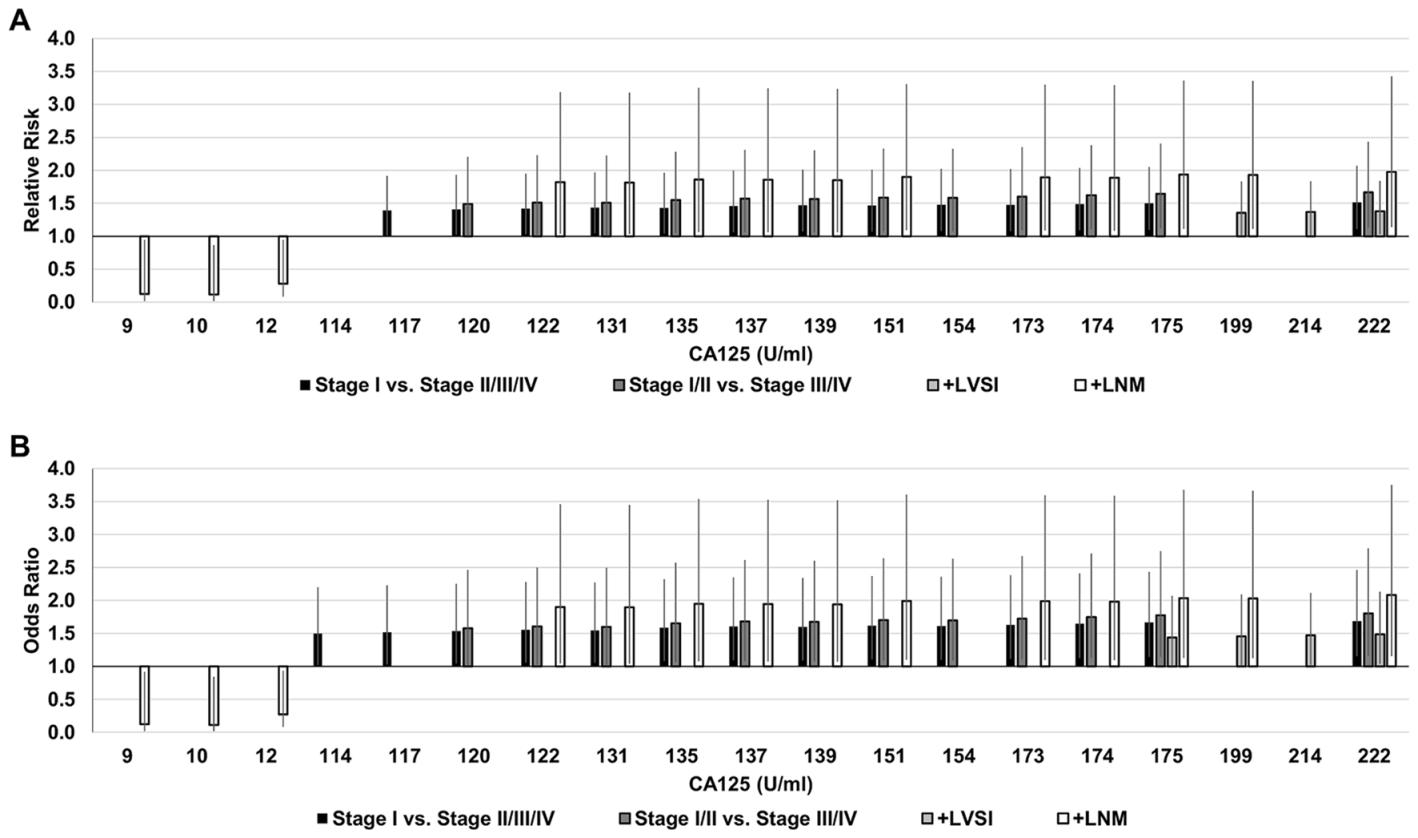
Relative risk (RR) (**A**) and odds ratio (OR) (**B**) of significant (*p* ≤ 0.05) CA125 thresholds. *P* values for OR and RR were calculated with Fisher's exact test using a two-sided 95% CI, where the CI was calculated using a normal approximation. Bars represent 95% CI.

RR and OR values that were found to be statistically significant are shown for all patient classifications in Fig. 5 The trends between OR and RR were the same. Patients having a CA125 of 9U/ml, 10U/ml, or 12U/ml had a decrease in risk of + LNM by 87, 88, and 72% respectively (0.13 fold, 0.12 fold, and 0.28 fold risk). This was the only decrease in risk that was significant. The largest increase in risk for patients having stage II/III/IV disease was 52% greater (1.52 fold risk) than for stage I while the largest increase in risk for patients having stage III/IV disease was 66% greater (1.66 fold risk), both at a CA125 level of 222U/ml or greater. The lowest CA125 threshold showing a significantly increased risk for stage II/III/IV (compared to stage I) and stage III/IV (compared to stage I/II) was 117U/ml and 120U/ml, respectively. Patients with a CA125 of 122U/ml or greater had a significantly increased risk of + LNM, with a maximum increase in the risk of 97% (1.97 fold risk) at a CA125 of 222U/ml. Patients with a CA125 of 199 U/ml or greater had a significantly increased risk of + LVSI, with a maximum increase in the risk of 38% (1.38 fold risk) at a CA125 of 222U/ml. No significant differences were found with respect to grade.

## Discussion

### Main findings

It is well known that improved preoperative risk stratification for EC is crucial due to the increased number of cases in the United States as well as the morbidity and mortality in high risk and advanced-stage disease. In the current analysis, we demonstrate that an elevated preoperative CA125 is associated with an increased risk of elevated stage, + LVSI, + LNM, and higher grade, all of which are associated with less favorable outcomes. In particular, this study demonstrates that a CA125 level of ≥ 222U/ml is associated with a 1.66 (1.14–2.44 95%CI) fold risk of having stage III/IV disease. Since a proportion of EC patients are first identified and some incidentally treated by general gynecologists, being able to risk-stratify patients preoperatively into low-risk versus high-risk is of utmost importance to ensure not only accurate patient referral to gynecologic oncologists but also to select appropriate surgical procedures.

### Strengths and limitations

In contrast to previous work, this study stratifies specific CA125 ranges at which there is an increase in relative risk of having a more advanced stage, higher grade, + LNM, and + LVSI. In this way, patients can be better risk-stratified in the preoperative period, allowing for more informed discussions regarding treatment options and having the improved knowledge of these risks during surgery. Previous studies have found that patients with deep myometrial invasion, + LVSI, or lymph node metastasis can have significantly elevated CA125. As such, CA125 levels are a marker in both preoperative and recurrent settings. If CA125 is elevated in the preoperative workup, it may be able to predict the need for further imaging, tests or biopsies prior to surgery, or better prepare patients for potential procedures to be incurred intraoperatively in the event of advanced malignancies.

Limitations of this study include a single-center retrospective study and lack of evaluation of a specific age associated with CA125. In addition, since elevated CA125 levels can occur in various benign gynecologic conditions and other disease states, such as leiomyoma and endometriosis, elevated CA125 may not be an ideal serum biomarker in premenopausal patients.

### Interpretation

Endometrial cancer is staged according to well defined and evidence-based surgical staging protocols, as clinical staging is less accurate and cannot assess LVSI, grade, or LNM. Proper surgical staging including lymph node assessment is crucial, especially in those with high-risk preoperative features to provide the best prognostic information. Previous studies have examined the predictive accuracy of high-risk features in endometrial cancer and serum markers such as CA125. Levels of CA125 ≥ 21.2 U/mL predicted + LVSI in women with EC in a study by Zhou et al.^17^. Logistic regression models were used in Yang et al.^18^ and Anton et al.^19^ to predict + LNM in EC patients. CA125 was found to be useful for predicting high-risk patients, including + LNM, by Zamani et al.^20^. Preoperative CA125 levels were significantly associated with deep myometrial invasion, advanced stage, and extrauterine and node metastasis in a study by Chen et al.^21^. Our study confirmed similar findings with a CA125 level of ≥ 222U/ml having up to a 38% (1.38 fold risk, 1.03–1.84 95%CI) increase in the risk of LVSI. Also, using the same level gives a significantly increased risk of + LNM with the maximum increase in the risk of 98% (1.98 fold risk, 1.14–3.43 95% CI) for a CA125 of ≥ 222U/ml. These data are further supported by the study by Antonsen et al.^22^, showing CA125 as a strong predictor for the presence of + LNM.

## Conclusion

Our study was able to stratify CA125 levels with respect to increases in stage, + LVSI, + LNM, and grade in patients with EC. Further, we were able to quantify specific values at which the risk for the presence of high-risk features in the preoperative patient with EC could be utilized to better risk-stratify patients before surgery. These data could also be used to minimize potential postoperative risks in patients who are poor surgical candidates. Evaluation of CA125 in relation to other potential clinical variables to enable a multivariable analysis will be considered in follow-on work.

## Methods

### Study design subjects and settings

This is a large single-center retrospective chart review of 890 patients diagnosed and surgically staged for EC between January 2000 to January 2015 at WellSpan York Hospital. Female patients over the age of 18 years old with a diagnosis of endometrial cancer and a pre-operative or within one week CA125 level were eligible for inclusion. All research was performed in accordance with the relevant institutional guidelines and regulations.

### Data collection

Data were abstracted using the PowerChart and E-care electronic medical records systems. Collected data included patient age at diagnosis, preoperative CA125 value, the procedure performed (total, subtotal or radical hysterectomy), both preoperative as well as postoperative histology, cancer grade (1/2/3), positive/negative lymph nodes, positive/negative lymphovascular invasion, pathologic stage (I/II/III/IV), pelvic/abdominal metastasis, race/ethnicity, physical exam findings, radiological imaging, and laboratory findings. The reference range of CA 125 is 0–35 units/mL (0–35 kU/L). The cutoff of 35 kU/L for CA 125 was determined from the distribution of values in healthy individuals to include 99% of the normal population^23^. CA 125 measurements were obtained with electrochemiluminescence Elecsys CA 125 II assay (Roche, Indianapolis, IN) and quantified by Labcorp (Burlington, NC). Currently, the standard method for assessing LVSI is light microscopic examination of haematoxylin and eosin (H&E) stained sections as the presence of tumor emboli within vascular channels lined by endothelial cells. The majority of patients had either endometroid adenocarcinoma (75.2%), serous (5.5%), mucinous (1.3%), clear cell (1.3%), mixed cell (4.2%) or carcinosarcoma (3.0%) histology, while the remainder had other histology subtypes (9.5%). Patients were subjected to either systematic lymphadenectomy or sentinel node sampling as determined by the operating physician in accordance with standard clinical protocol. Both pre-operative and post-operative pathology were analyzed by the same clinical oncology/pathology team.

### Exclusion criteria

Patients with a historical diagnosis of endometrial cancer, those who did not have a documented CA1-25 level, pregnant women, or those who had been previously staged surgically were excluded from the study.

### Outcome variables

The primary outcome was to determine OR and RR between a preoperative CA125 level and the likelihood of lymph node metastases, lymphovascular invasion, advanced stage, and high grade. Based on CA125 levels, a secondary outcome was to identify CA125 ranges that correspond to these variables.

### Statistical analysis

Data analyses were conducted in the R programming language (version 4.0.2)^24^, with scripts that utilized functions from the following R packages: Odds ratios (OR) and relative risk (RR) were calculated by unconditional maximum likelihood estimation (Wald) using *oddsratio* and *riskratio* functions from *epitools* package^25^. In this retrospective study, both RR and OR were calculated to improve data interpretation and avoid exaggerated estimates, as OR has the possibility to overestimate event odds when there is a relationship between patient groups and outcomes^26^. For CA125 averages and medians, 95% confidence intervals were produced with a non-parametric bootstrap method using *boot* function from the *boot* package where the number of resampling iterations was 1000 and the statistic was the average or median, respectively. *P* values for comparing medians and means were calculated using Wilcoxon rank-sum test and unpaired student’s T-test, respectively. *P* values for OR and RR were calculated with Fisher's exact test using a two-sided 95% CI, where the CI was calculated using a normal approximation.

### Data classification

OR and RR were determined for CA125 levels as the predictor with staging, grade, + LVSI, and + LNM as outcomes. All RR and OR values were calculated relative to a CA125 level of 35U/ml or below being considered normal. Staging was subclassified as follows: stage I versus stage II/III/IV; stage I/II versus stage III/IV; stage I/II/III versus stage IV. Grade was subclassified as follows: grade 1 versus grade 2/3; grade 1/2 versus grade 3. CA125 thresholds were incremented by 1U/ml from 1 to 222 with OR and RR re-calculated at every threshold. A CA125 level of 35 U/ml was used as a baseline, as values at or below this level are considered normal.

### Ethics approval

The study was approved by Institutional Review Board (IRB) at York Hospital Human Subjects Research Protection Program Office (IRB number 731831-1). The study was exempt from informed consent.

## Data Availability

The datasets used and analyzed during the current study are available from the corresponding author upon reasonable request.

## Abbreviations

RR: Relative risk
CA125: Cancer antigen 125
EC: Endometrial cancer
FIGO: International Federation of Gynecology and Obstetrics
MI: Myometrial invasion
DVT: Deep vein thrombosis
LVSI: Lymphovascular space invasion
+LVSI: Positive LVSI diagnosis
LNM: Lymph node metastasis
+LNM: Positive LNM diagnosis
